# Evaluating the causes of retinopathy of prematurity relapse following intravitreal bevacizumab injection

**DOI:** 10.1186/s12886-024-03528-0

**Published:** 2024-06-21

**Authors:** Amir Eftekhari Milani, Amin Arasteh, Zahra Saeedi-Maleki, Mohamad Reza Niyousha, Mohamad Ali Sahebazamani, Fariborz Brumandpur

**Affiliations:** 1 Department of Ophthalmology, Nikookari Eye Hospital, Tabriz University of Medical Sciences, Tabriz, Iran; 2 Student Research Committee, Tabriz University of Medical Sciences, Tabriz, Iran

**Keywords:** Bevacizumab, Recurrence, Retinopathy of prematurity, Risk factors

## Abstract

**Background:**

Retinopathy of prematurity (ROP) is a proliferative disorder of the developing retina. Intravitreal bevacizumab injection (IVB) is an emerging treatment for severe forms of ROP, which does not restrict the visual field in comparison to laser therapy. The present study aimed to determine and evaluate the risk factors for ROP recurrence following IVB injection.

**Materials and methods:**

In this retrospective study, 98 eyes of 49 infants with ROP who had received IVB injections as the primary treatment for type 1 ROP are included.

**Results:**

Fifty-four eyes (55.1%) had aggressive retinopathy of prematurity (A-ROP), and forty-four (44.9%) had Stage III Plus ROP in Zone II. ROP recurred in 13 eyes (13.26%) of 8 infants. The mean period between IVB and the ROP recurrence was 8.08 (95% CI:5.32–10.83) weeks. The infants who had ROP recurrence had lower birth weight (P value = 0.002), lower postmenstrual age at IVB injection (P value = 0.001), lower IVB injection gap period from birth (P value = 0.044), higher oxygen therapy requirement rate after IVB injection (P value < 0.001, OR:19.0) and higher oxygen therapy duration (P value = 0.006). The ROP severity, gestational age at birth, and diet were not statistically different between the recurrence and complete regression groups. Out of 13 eyes treated with laser photocoagulation because of ROP relapse, macula dragging occurred in one eye, and all the cases met the complete regression.

**Conclusion:**

Low birth weight and oxygen therapy are the most important risk factors for ROP relapse, which requires meticulous oxygen treatment guidelines for premature infants.

## Introduction

Retinopathy of prematurity (ROP) is one of the major causes of potentially avoidable blindness among infants worldwide [1]. Blood levels of oxygen and their fluctuations have a prominent role in the physiopathology of ROP in preterm neonates by affecting endothelial growth factor secretion. Coincidence of other morbidities such as respiratory failure, sepsis, poor nutrition and weight gain, and blood sugar fluctuations could exacerbate the risk of ROP in premature infants [2]. The incidence of ROP is constantly increasing as more immature infants survive due to the improvement of neonatal care [3]. Laser photocoagulation is currently the gold standard treatment for ROP but might restrict the visual field and contribute to myopia development [4, 5]. The BEAT-ROP study demonstrated a beneficial effect for intravitreal Bevacizumab (IVB) vs. laser in treating Zone I, Stage 3 + ROP [6]. IVB has also been used safely for the treatment of aggressive ROP (A-ROP) [7]. IVB treatment could rapidly lead to the regression of vascular abnormality, especially in eyes with miotic pupils, and also lower the incidence of induced high myopia in type 1 ROP, compared with laser ablation. However, recurrence remains a major concern in the administration of IVB [8]. Different risk factors have been related to ROP recurrence, such as Zone I ROP, early need for treatment, and low Apgar score [9]. In this study, we aimed to retrospectively investigate children who received IVB as the primary treatment of ROP to compare some risk factors between those with and without ROP recurrence and complications after ROP recurrence treatment.

## Materials and methods

### Patients and examinations

This retrospective study investigated ROP recurrence risk factors among those who received IVB because of type 1 ROP during one year (September 2022 to September 2023) in Nikookari Hospital, Tabriz, Iran. This center is the referral ROP center in the northwest of Iran.

In this study, 98 eyes of 49 infants with ROP who had received IVB injections as the primary treatment are included. The exclusion criteria were IVB complications such as endophthalmitis and cataract formation. We did not have any infants with exclusion criteria. All of the patients had regular follow up at scheduled times. Recurrence was defined as the redevelopment of plus disease, pathological new vessels, or elevated ridge following a complete regression of ROP after IVB injection. We compared the following risk factors between those with and without ROP recurrence: gestational age (GA) (weeks), postmenstrual age at IVB injection time (days), IVB injection gap period from birth (days), Birth weight (BW) (grams), any history of oxygen therapy after IVB injection, mean oxygen therapy period after IVB injection (days), ROP Stage, and diet (breastfeeding, powdered milk or mixed). A retina subspecialist examined all infants after pupillary dilation with topical tropicamide 0.8%. Indirect funduscopy by pan-retinal Volk lens (Mentor, Ohio 44,060, US) was used for retinal examination. The same physician examined all patients during follow-up visits and the IVB injections. The IVB was injected on the same day of the examination for type 1 ROP.

Any ROP stage with plus disease in zone I, stage 3 ROP without plus disease in zone I, and stage 2 or 3 ROP with plus disease in zone II are classified as type 1 ROP [10]. In the case of ROP recurrence, the same physician applied retinal laser photocoagulation.

### Intravitreal injections

The parents of all the neonates were informed about the IVB injection procedure and its possible side effects and complications. Then, written informed consent forms were taken before the procedure. All the neonates received intravitreal Bevacizumab (0.25 mg/0.01 mL) injection with a gauge-30 needle inserted at 0.5 to 1.0 mm distance from limbus supra-temporally under sedation and topical anesthesia.

### Ethical considerations

The study was approved by the medical research ethics committee at Tabriz University of the Medical Sciences with an approval code of IR.TBZMED.REC.1401.800. Due to the study’s retrospective nature and the presence of a general informed consent form for medical data usage in medical research for all admitted patients, the research ethics committee waived obtaining personal informed consent forms from each patient and their parents for participating in this study.

### Statistical analysis

All the statistical analysis in this study was performed using IBM SPSS Statistics 27.0, and the Kaplan-Meier curves were depicted by GraphPad Prism 10.0. The distribution of the quantitative data was examined by the Kolmogorov-Smirnov test and histograms. The normally distributed variables were analyzed by parametric tests such as the student’s T-test, and the other data were analyzed by non-parametric tests such as Mann-Whitney U. The categorical variables were analyzed by Chi-square and Fisher-Freeman-Halton Exact Test. The alpha error in this study is considered 0.05, and p-values lower than 0.05 are considered statistically significant.

## Results

### Recurrence rate and ROP severity

**Fig. 1:**
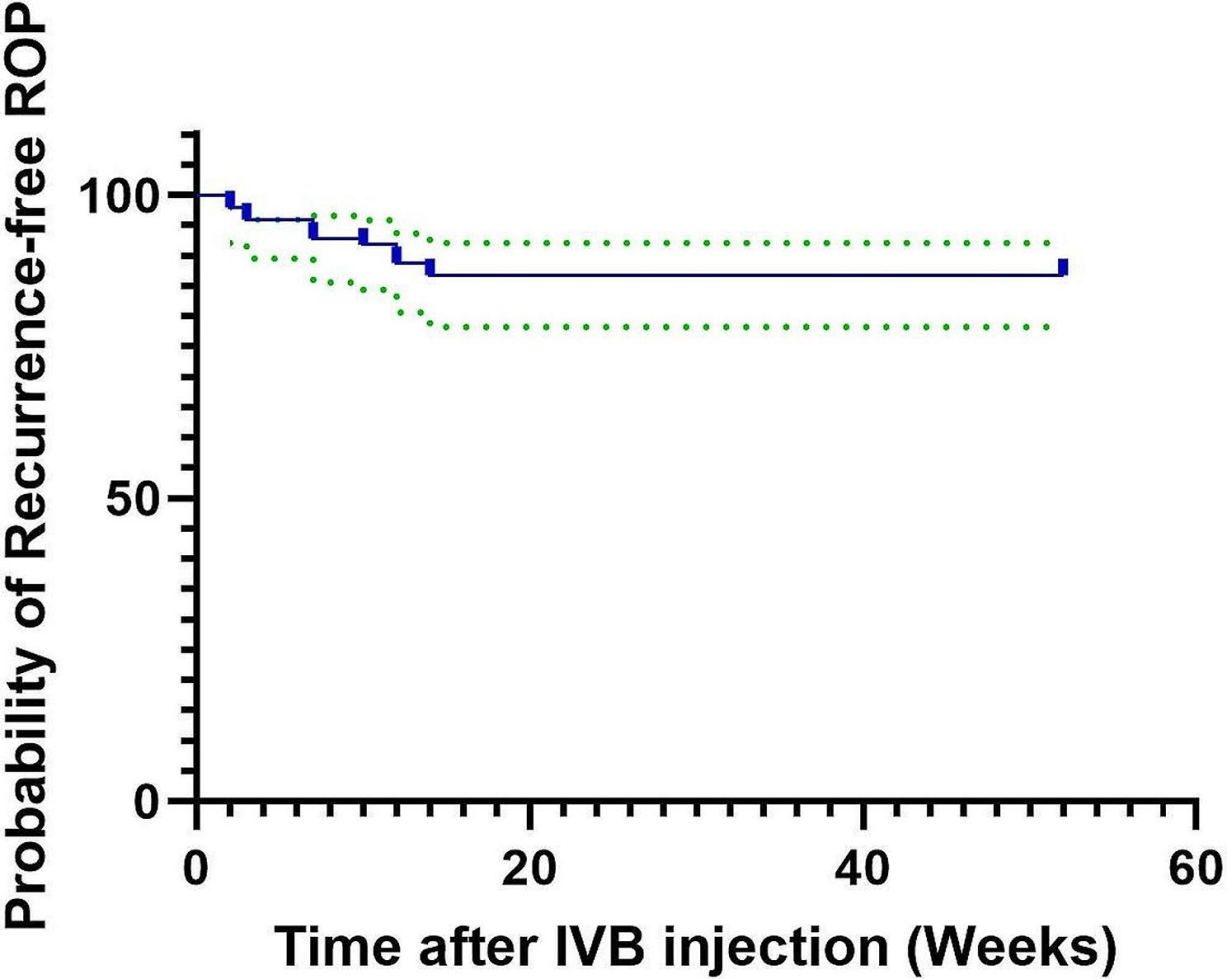
Kaplan-Meier recurrence-free probability curve of ROP received IVB. The blue line demonstrates the probability of recurrence-free condition, and the green lines demonstrate the 95% confidence interval

98 eyes of 49 infants with ROP who had received intravitreal Bevacizumab are included in our study. 55.1% of the ROP cases had A-ROP, and 44.9% had Stage III Plus ROP in Zone II. Despite the intravitreal injection, the ROP recurred in 13 eyes (13.26%) of 8 infants. The mean period between the IVB injection and the ROP recurrence was 8.08 (95%CI:5.32–10.83) weeks. Figure 1 shows the Kaplan-Meier curve of ROP recurrence after IVB injection. All the eyes with ROP recurrence received laser photocoagulation therapy, and 12 achieved anatomic improvements and ROP regression without complications. However, in one eye, macular dragging happened after laser therapy and ROP regression.

**Fig. 2:**
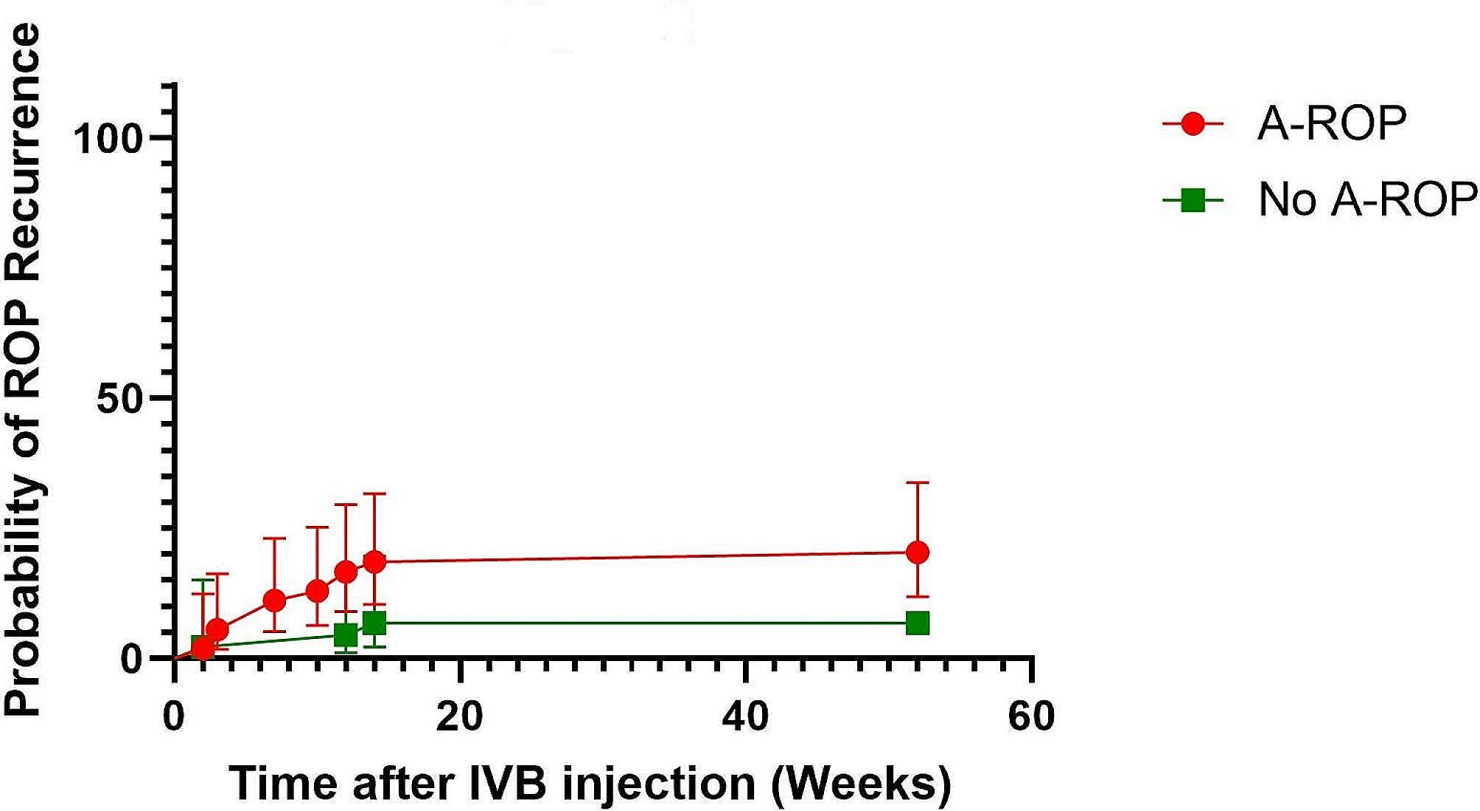
Kaplan-Mier curve of ROP recurrence in two groups regarding the presence of A-ROP before IVB injection. The red points and line demonstrate the ROP recurrence probability in the A-ROP cases, and the green line shows the other group. Bars show the 95% confidence interval. The A-ROP cases seem to have a higher probability of ROP recurrence after IVB injection

76.9% of the infants with ROP recurrence had A-ROP before IVB injection; on the other hand, the prevalence of the A-ROP in the infants with complete ROP regression was 51.7%. Although the recurrence rate in the A-ROP group was 18.51% compared to 6.81% in the zone II ROPs, this difference was not statistically significant regarding the Chi-Square test (p-value = 0.089). The remaining infants in both groups had Stage III Plus ROP in Zone II. Although the survival analysis of the A-ROP cases versus not A-ROP cases did not show a statistically significant difference between the two groups (p-value = 0.057), the Kaplan-Mier curve of ROP recurrence (Fig. 2) seems to demonstrate a higher rate of ROP recurrence in A-ROP cases.

The A-ROP prevalence was also compared between the recurrence and complete regression cases of infants who received supplemental oxygen after IVB injection; Fisher’s exact test results did not show any significant difference (p-value = 0.131). The multivariate logistic regression modeling also failed to show any significant role for A-ROP’s presence in the ROP recurrence prediction (p-value = 0.471). These statistically insignificant results may be due to the limited sample size.

### Gestational age and birth weight

The median GA of the infants with the ROP recurrence was 26 (the interquartile range (IQR): 26.00–29.00) weeks at birth. It was 29 (IQR: 27.25-30.00) weeks for the infants without the ROP recurrence. According to the Independent-Samples Mann-Whitney U Test, the two groups have no statistically significant difference (p-value = 0.072). On the other hand, the ROP recurrence group received the IVB injection at the median postmenstrual age of 232 (IQR: 229.50-244.50) days compared to 251 (IQR: 245.00-257.00) days in the regressed eyes, which is a statistically significant difference (p-value = 0.001). The ROP recurrence cases received the IVB injection in younger gestational ages compared to the complete regression cases. Also, when the time gap between the birth and the IVB injection was compared, results showed a significantly shorter gap period for the ROP recurrence cases. Completely regressed ROP cases received the IVB injection in a median of 50 (IQR: 40.00–60.00) days from birth, while the recurrence cases received 30 (IQR: 30.00-58.50) days after birth (p-value = 0.044).

In addition, the two groups significantly differed regarding the BW. The median weight for infants with the ROP recurrence was 1000 (IQR: 732–1000) grams versus 1100 (IQR: 1000–1350) grams in the other group (p-value = 0.002). The infants who achieved the ROP regression without recurrence after the IVB injection had higher BW.

### Supplemental oxygen therapy

**Fig. 3:**
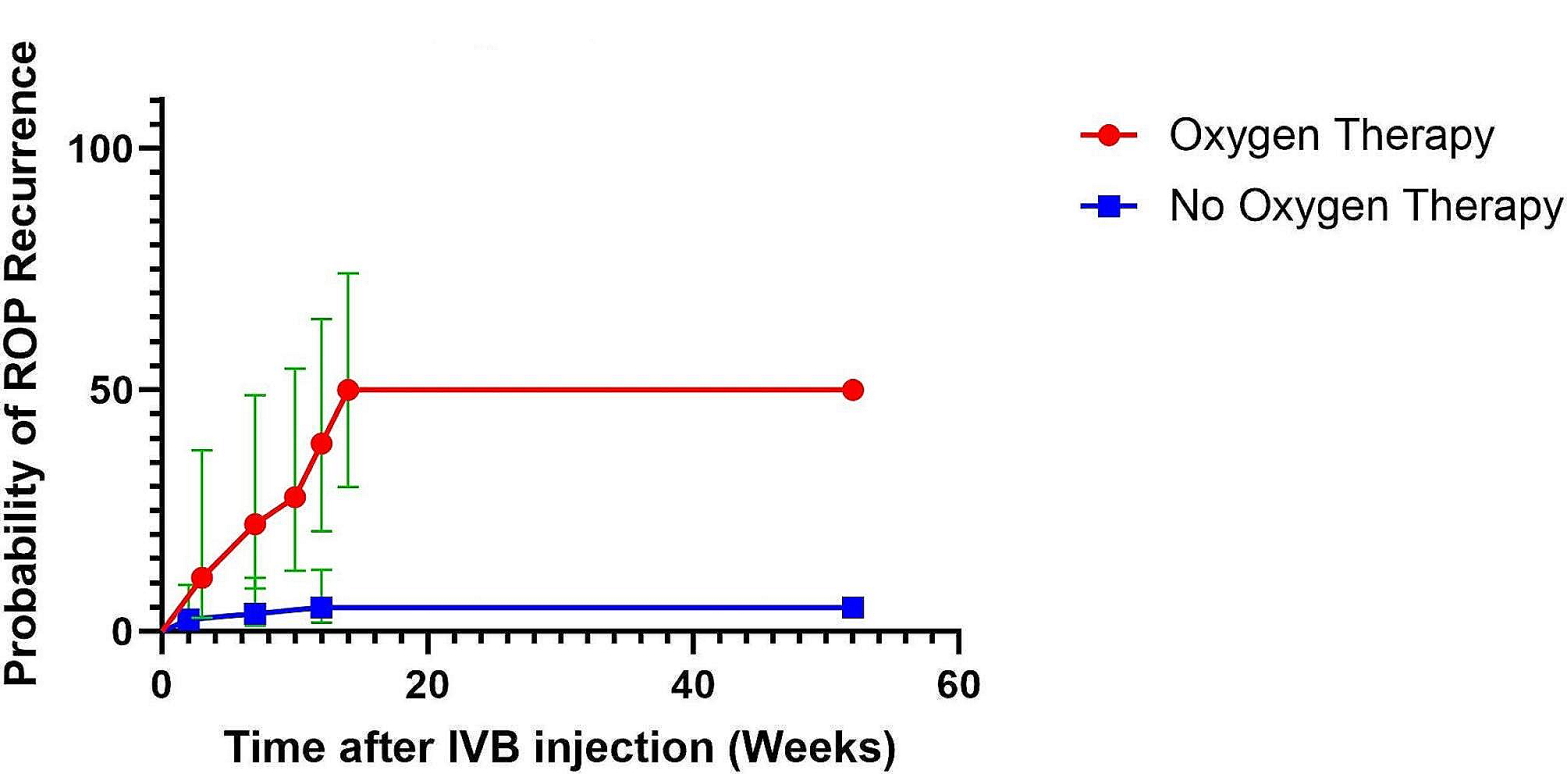
Kaplan-Mier curve of ROP recurrence in two groups regarding the need for supplemental oxygen therapy after IVB injection. The red points and line demonstrate the ROP recurrence probability in the group receiving supplemental oxygen therapy, and the blue line shows the other group. Bars show the 95% confidence interval. The group in need of supplemental oxygen therapy after IVB injection has a significantly higher risk of ROP recurrence

10.58% of completely regressed ROP cases needed supplement oxygen therapy after the IVB injection compared to 69.23% of those in the ROP recurrence group. The difference between the two groups is statistically significant (p-value < 0.001). The odds ratio for supplemental oxygen therapy after IVB injection is 19.0 (95%CI: 4.8–74.4). While the prevalence of supplemental oxygen therapy was higher in the ROP recurrence group, there was also a significant difference in the mean oxygen therapy period between infants who received oxygen in the two groups (p-value = 0.006). The mean period of oxygen therapy after the IVB injection was 9.0 (95%CI: 6.5–11.4) days in the ROP recurrence cases and 5.0 (95%CI: 5.00–5.00) in the complete regression group. The ROP recurrence cases, on average, received supplemental oxygen for 4.0 (95%CI: 1.5–6.4) days more than the other group after the IVB injection. Figure 3 shows the Kaplan-Meier curve of ROP recurrence in two groups regarding the need for supplemental oxygen therapy after IVB injection.

### Diet

The two groups had no significant difference in the infants’ diet. 51.8% of wholly regressed ROPs received a combination of maternal and powdered milk, and 61.6% in the ROP recurrence group had the same diet (p-value = 0.097). Neither the maternal nor powdered milk was associated with the ROP recurrence (p-value = 0.321 and p-value = 0.064, respectively). The ocular and demographic characteristics of the infants are available in Table 1 in more detail.

**Table 1:** Ocular and demographic characteristics of infants with ROP in two groups of complete regression and recurrence after IVB injection. T: Students’ T-test, M: Mann-Whitney U, C: Chi-square, F: Fisher-Freeman-Halton Exact Test

		ROP Recurrence (*n*:13)	ROP Regression (*n*:85)	*p*-value
Gestational age (weeks)		26 (IQR: 26.00–29.00)	29 (IQR: 27.25-30.00)	0.072^M^
Postmenstrual age at IVB injection (days)		232 (IQR: 229.50-244.50)	251 (IQR: 245.00-257.00)	**0.001** ^**M**^
IVB injection gap period from birth (days)		30 (IQR: 30.00-58.50)	50 (IQR: 40.00–60.00)	**0.044** ^**M**^
Body weight (grams)		1000 (IQR: 732–1000)	1100 (IQR: 1000–1350)	**0.002** ^**M**^
Oxygen therapy after IVB injection (n)		9 (69.23%)	9 (10.58%)	**< 0.001** ^**C**^
Mean oxygen therapy period (days)		9.0 (95%CI: 6.5–11.4)	5.0 (95%CI: 5.00–5.00)	**0.006** ^**T**^
Stage (n)	A-ROP	10 (76.9%)	44 (51.7%)	0.089^C^
	Z(II) S(III) plus	3 (23.1%)	41 (48.3%)	
Diet (n)	Maternal	0 (0%)	20 (23.5%)	0.097^F^
	Powdered	5 (38.4%)	21 (24.7%)	
	Mixed	8 (61.6%)	44 (51.8%)	

## Discussion

The physiological process of human embryonic retinal vascularization consists of vasculogenesis and angiogenesis. Through vasculogenesis, blood vessels are generated by the endothelial cells’ differentiation from their progenitors. This process takes place between the 12th and 21st week of gestational age. Angiogenesis is the process that leads to the formation of the superficial plexus. During angiogenesis, the retinal capillary plexus is generated from the optic nerve retinal vessels, branched and elongated towards the peripheral retina. These new vessels reach the nasal and temporal periphery at 32 and 36–40 weeks of gestational age, respectively [10].

ROP is a vasoproliferative disease caused by Vascular endothelial growth factor (VEGF) production due to retinal ischemia. IVB has been suggested as a treatment for ROP in the presence of aggressive posterior ROP, miotic pupil, or media opacity. Its advantages over laser therapy are its availability, simple injection technique, and visual field preservation [11]. It has some disadvantages compared to laser therapy, including endophthalmitis, cataract formation, and a higher recurrence rate, especially after a more extended period [9]. The major risk factors for ROP are prematurity (low GA and birth weight) and supplemental Oxygen therapy. More advanced neonatal care and specialized hospitals could reduce the ROP incidence rate, as small hospital studies compared with tertiary referral hospitals approve of it [12]. When the GA is lower, the area of the ischemic retina is more extensive. As a result, the concentration of VEGF in the vitreous cavity would be higher, so the probability of type 1 ROP requiring treatment would be higher [13].

ROP recurrence is the redevelopment of plus disease, pathological new vessels, or elevated ridge following a complete regression of ROP following treatment [14]. The recurrence rate in our study was 13.26%, which was relatively lower than in similar studies. A study comparing laser photocoagulation and intravitreal Ranibizumab injection showed an 18.3% recurrence rate in the Ranibizumab group. However, the mean interval between the injection and recurrence was similar (8.08 vs. 9.3 weeks) [15]. On the other hand, another study showed a much lower recurrence rate (4.04%) after IVB injection in ROP cases. This lower recurrence rate might be related to the lower prevalence of Zone I ROP (21.6%) in the sample size [8]. Although the distribution of the various stages of ROP did not show any statistical difference between the two groups in our study (A-ROP prevalence 76.9% vs. 51.7% in recurrence and complete regression cases, respectively), this statistically insignificant result may be due to the small sample size and more advanced stages of ROP may increase the recurrence rate. It should also be mentioned that the study took place in the referral center of ROP in the northwest of the country, where most advanced and complicated cases have been compared to other ophthalmology centers. Regarding this fact and inclusion of IVB-treated cases in the study, the A-ROP cases prevalence is higher in this study compared to the typical ROP population (about 5.5%) [16].

In our study, low birth weight was a risk factor for ROP recurrence. However, lower GA was not statistically significant (the median GA was 26 weeks in the group with ROP recurrence compared to 29 weeks in those without recurrence). This statistically insignificant difference can be due to the study’s low recurrence rate and small sample size. Most studies have confirmed that lower GA and weight are risk factors for ROP and its recurrence [7–9]. However, logistic regression in a large study revealed that the GA and Birth weight are not independent risk factors for the recurrence after Ranibizumab [15]. The possible relationship between lower GA and Birth weight with ROP recurrence can be explained by more immature retinal vasculature, which leads to a larger area of ischemic retina, as mentioned above. Although GA was not a statistically significant risk factor for recurrence in our study, the lower postmenstrual age at IVB injection was associated with ROP recurrence. The Iwahashi et al. showed a higher rate of recurrence in neonates receiving anti-VEGF threpy earlier than 35-week of postmenstrual age [17].

Supplemental oxygen therapy has been described as a risk factor for ROP development [2, 12, 18]. In this study, we investigated the role of oxygen therapy after IVB injection. The number of children who received Oxygen after IVB injection and the duration of oxygen therapy were higher in the group with ROP recurrence. Similar to our study, the study by Ling et al. showed that oxygen therapy after IVB injection or intravitreal ranibizumab injection was associated with higher ROP relapse [9]. It has been declared that O_2_ saturation fluctuations are more related to higher oxidative stress and ROP occurrence than steady prolonged hyperoxia [19]. So, infants who need oxygen therapy even after IVB injection may have more unstable respiratory and metabolic conditions, leading to more oxidative stress, which induces ROP recurrence.

Besides, we have to take into account that these children are hospitalized due to respiratory problems or other systemic conditions that can worsen retinal hypoxia or ischemia. Premature infants are more prone to other exacerbating conditions, such as infections, and may have more severe forms of retinopathy due to the lower compliance of anti-oxidant metabolic pathways [8]. With lower oxygen saturation, the rate of mortality increases; with higher oxygen saturation, the rate of ROP increases [20]. So, it is hard to determine which oxygen saturation level suits these patients.

Similar to Ling et al. study [9], those infants who received IVB injection sooner after birth had a higher probability of ROP recurrence (the median of 30 days in those with ROP recurrence versus 50 days in those without relapse). This difference shows that the imbalance between angiogenic and anti-angiogenic agents occurs at a shorter period in these children, leading to the development of type 1 ROP. So, the probability of reaching this unbalanced state in the future after IVB injection is higher among these infants.

Recent studies hypothesized that lower Insulin-like growth factor-1 (IGF-1) levels at birth could be related to the ROP occurrence, and infants’ nutrition could alter the IGF-1 levels [21]. In addition, a systematic review demonstrated a protective role for human milk intake against the ROP and its severe forms [22]. We aimed to find any possible association between the infants’ diet type and ROP recurrence, which is not addressed well in the literature; however, we did not find any significant relationship.

Although our findings could help to clarify some of the risk factors for ROP recurrence after IVB therapy, like every study, it had some limitations. Our study was retrospective and was conducted in the eye hospital, so we did not have access to the medical documents of cases in pediatric hospitals. This issue made us unable to evaluate the role of other significant risk factors, such as a history of sepsis, anemia, and blood transfusion. So, we could not control some of the confounding factors mentioned. Besides, some statistical analyses were not possible due to limited cases of ROP recurrence. More extensive prospective studies with more comprehensive data gathering would be more fruitful in addressing the research gap.

## Conclusion

ROP is the most common cause of visual impairment among infants. So, the most critical point in ROP management is preventing severe forms (type 1) that require treatment by improving neonatal care and well-established ophthalmic screenings for preterm infants. Although IVB injection could be a suitable replacement for laser photocoagulation in the ROP treatment, the recurrence after treatment should be considered, and infants should be monitored closely, especially until ten weeks, when most recurrences occur. Infants with lower Birth weight, an earlier need for IVB injection, and those who need supplemental oxygen for a more extended period should be considered at high risk for recurrence. Besides, a collaboration between neonatologists and ophthalmologists could lower the recurrence rate by minimizing post-IVB oxygen therapy.

## Data Availability

The datasets used and or analysed during the current study are available from the corresponding author on reasonable request.

## Abbreviations

ROP: Retinopathy of prematurity
IVB: Intravitreal bevacizumab injection
A-ROP: Aggressive retinopathy of prematurity
VEGF: Vascular endothelial growth factor
GA: Gestational age
IGF-1: Insulin-like growth factor-1
BW: Body weight
